# New distributional records for sixteen Mordellidae species from the Western Palearctic (Insecta, Coleoptera, Mordellidae)

**DOI:** 10.3897/zookeys.894.39584

**Published:** 2019-12-04

**Authors:** Dávid Selnekovič, Enrico Ruzzier

**Affiliations:** 1 Department of Zoology, Faculty of Natural Sciences, Comenius University in Bratislava Ilkovičova 6, SK-84215, Bratislava, Slovakia Comenius University Bratislava Slovakia; 2 World Biodiversity Association Onlus, c/o Museo Civico di Storia Naturale, Lungadige Porta Vittoria 9, Verona, Italy World Biodiversity Association Onlus Verona Italy

**Keywords:** Bionomy, Bosnia and Herzegovina, Bulgaria, Cyprus, distribution, faunistics, host plants, Italy, Kazakhstan, Kyrgyzstan, Montenegro, *
Mordellistena
*, *
Mordellochroa
*, Morocco, North Macedonia, Slovakia, Spain

## Abstract

A list of 22 new distributional records is presented for 16 Mordellidae species from the Western Palearctic: *Variimorda caprai* (Franciscolo, 1951) (Montenegro); *V. mendax* Méquignon, 1946 (Montenegro); *Mordellistena falsoparvula* Ermisch, 1956 (Bosnia and Herzegovina, Montenegro); *M. olympica* Ermisch, 1965 (Cyprus, Montenegro); *M. kraatzi* Emery, 1876 (Morocco); *M. longicornis* Mulsant, 1856 (Morocco); *M. dives* Emery, 1876 (Kazakhstan); *M. krujanensis* Ermisch, 1963 (Montenegro); *M. tarsata* Mulsant, 1856 (Cyprus, North Macedonia); *M. michalki* Ermisch, 1956 (Kyrgyzstan); *M. thuringiaca* Ermisch, 1963 (Bulgaria, Montenegro, Slovakia, Spain); *M. koelleri* Ermisch, 1956 (Italy, Montenegro); *Mordellistenula longipalpis* Ermisch, 1965 (Montenegro); *Mordellochroa milleri* (Emery, 1876) (Italy); *Dellamora palposa* Normand, 1916 (Italy). Information about the distributional range is summarised for each species, and notes on habitat and host plants are also provided.

## Introduction

Mordellidae Latreille, 1802 is a rather diverse but poorly known and sporadically studied family of beetles. It comprises more than 2300 described species distributed nearly worldwide except for the polar and subpolar zones. Mordellid beetles inhabit various ecosystems including tropical rainforests, temperate deciduous forests, grasslands and ruderal ecosystems. The majority of species are pollinivorous in adult stages, feeding on a wide variety of plant species. Exceptions are the members of the genus *Glipa* Leconte, 1859, reported to feed on fern spores (Takakuwa 2000), or the South American *Boatia albertae* Franciscolo, 1985 which was found to have the foregut filled with fungal spores (Lawrence and Ślipiński 2010). Larvae of some mordellid beetles are wood borers (e.g., *Mordella* Linnaeus, 1758, some *Mordellistena* Costa, 1854), the others develop in sporocarps of Polyporaceae fungi (e.g., *Curtimorda* Méquignon, 1946, *Mordella marginata* Melsheimer, 1845), or in stems of various herbaceous plants (e.g., *Mordellistena* Costa, 1854, *Mordellistenula* Shchegoleva-Barovskaya, 1930). In some cases, mordellid larvae seem to have predaceous habits against other insect larvae (Tooker and Hanks 2004) or can be inquilines of termite colonies (Hill 1922).

The majority of distribution records available on Palearctic Mordellidae are usually included in publications focused primarily on the taxonomy (e.g., Ermisch 1963b, 1965, 1977; Horák 1985; Plaza 1985). Several authors have summarised the distributional information on regional level (e.g., Ermisch 1956, 1963a, b, c, 1969b, 1977; Köstlin and Vogt 1971; Batten 1976b; Franciscolo 1995; Odnosum 2003, 2010; Zemoglyadchuk 2007; Ruzzier 2013; Ruzzier et al. 2017). Comprehensive catalogues were published, for instance, by Heyden et al. (1906), Csiki (1915), and Horák (2008). Despite the great effort of these authors, the knowledge of the distribution of many Palearctic species can be still considered poor, and several species remain to be known only from the localities stated in the original description.

Herein we provide new distributional and bionomical data obtained during the recent collecting activities of both authors (2010–2018) and by the re-examination of the material deposited in museums or private collections listed below. We also summarise the previously published distributional data and provide the lists of countries from which the species have been previously reported.

## Materials and methods

The present study is based on the material sampled by the authors or accessed from the museums and private collections listed below. Specimens were collected individually from flowers or by sweeping the vegetation. Larvae of *Mordellistena* species were reared from stems of herbaceous plants collected in April, cut to the smaller pieces (ca. 20 cm), transferred to the laboratory, and stored in plastic containers with air access. Adult specimens were killed in ethyl acetate. Dry specimens were relaxed in water with a small amount of acetic acid, then dissected and glued on cards. Dissected genitals were glued with the respective specimen using dimethyl hydantoin formaldehyde (DMHF) or stored in micro-vials containing glycerol and pinned under the specimen. Habitus images were taken by Cannon 5D mark IV attached to Zeiss Axio-Zoom V-16 stereomicroscope with diffuse LED lighting, stacked in Zerene Stacker 1.04 and subsequently edited in Adobe Photoshop CC.

Identifications were carried out using original species descriptions, identification keys (e.g., Ermisch 1956, 1963b, 1969a, 1977) or by comparison with the type material. Each specimen was labelled with identification data containing full species name, name of the identifier and year when the identification was made. Examined specimens are deposited in the following collections:

**DSPC** Dávid Selnekovič private collection, Bratislava, Slovakia

**EEPC** Eduard Ezer private collection, Zlín, Czech Republic

**ERPC** Enrico Ruzzier private collection, Mirano, Italy

**HNHM** Hungarian Natural History Museum, Budapest, Hungary

**SNMB** Museum of Natural History, Slovak National Museum, Bratislava, Slovakia

**SNSD** Senckenberg Naturhistorische Sammlungen, Dresden, Germany

In the section “New records”, we provide the data on examined material concerning only those countries from which the species are reported for the first time. The “Distribution” section contains the list of countries from which the species have been previously reported, followed by the citation of its first appearance in the published sources. In the “Remarks” section, we provide information about habitat preferences, host plants and collecting circumstances (if available).

## Results

In the list below, we present 22 new country-level records of 16 Mordellidae species. The list is based on 201 examined specimens from thirty localities in the Western Palearctic. We also provide information about distribution and bionomy for each species.

### Variimorda (Galeimorda) caprai

Taxon classificationAnimaliaColeopteraMordellidae

(Franciscolo, 1951)

8C4F8562-3EF0-599D-91E0-C5DCB73716D8

Fig. 1A

Mordella (s. str.) caprai Franciscolo, 1951: 7–9 [type locality: Shkodër, Albania].
Variimorda caprai : Ermisch 1969b: 846, 849.
Variimorda (Galeimorda) caprai : Horák 1985: 15.

#### New records.

MONTENEGRO • 43 ♂♂, 13 ♀♀; Bar env., Volujica hill; 42°04'29.0"N, 19°06'11.8"E; 20 June 2011; D. Selnekovič leg.; slopes with dry grassland vegetation; on the flowers of *Helichrysum*; D. Selnekovič det.; DSPC • 15 ♂♂, 12 ♀♀; Bar env., Ribnjak Monastery; 42°07'56"N, 19°07'33"E, 22 June 2011; D. Selnekovič leg.; slopes with dry grassland vegetation; on the flowers of *Helichrysum*; D. Selnekovič det.; DSPC.

#### Distribution.

Albania (Franciscolo 1951), Montenegro (**new country record**).

#### Remarks.

*Variimorda caprai* was described based on three male specimens from Shkodër, Albania and since then no other record has been published. In 2011, the first author collected 83 specimens in the environment of Bar in Montenegro on the slopes with xerothermophilous grassland vegetation (Fig. 2). Specimens were found feeding on the flowers of *Helichrysum*. The immature stages and their host plants remain unknown.

**Figure 1.**
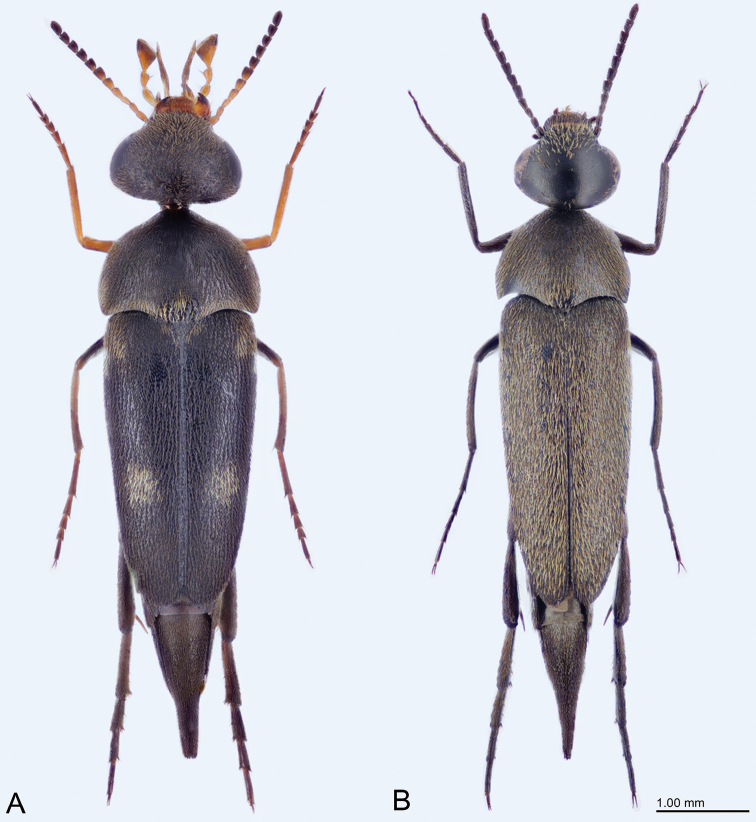
**A**Variimorda (Galeimorda) caprai (Franciscolo, 1951), male **B**Mordellistena (s. str.) dives Emery, 1876, male.

### Variimorda (s. str.) mendax

Taxon classificationAnimaliaColeopteraMordellidae

Méquignon, 1946

8B772C2F-1D80-5DAC-B3F2-23CE723BA84C

Mordella (Variimorda) mendax Méquignon, 1946: 63, 71–72 [type locality: Laigneville, France].
Mordella (Variimorda) mendax var. devillei Méquignon, 1946: 71–72 [type locality: Bordeaux, France].
Mordella (Variimorda) mendax var. chobauti Méquignon, 1946: 71–72 [type locality: La Bonde, France].
Variimorda (s. str.) mendax : Ermisch 1956: 277.

#### New records.

MONTENEGRO • 1 ♂; Bar; 42°06'36"N, 19°05'20"E; 19 June 2011; D. Selnekovič leg.; ruderal vegetation in urban environment; on the flowers of *Daucus carota*; D. Selnekovič det.; DSPC.

#### Distribution.

Albania (Ermisch 1969b), Algeria (Méquignon 1946), Austria (Méquignon 1946), Azerbaijan (Ermisch 1956), Bosnia and Herzegovina (Ermisch 1956), Bulgaria (Ermisch 1969b), Croatia (Ermisch 1956), Czech Republic (Méquignon 1946), France (Méquignon 1946), Georgia (Horák 2008), Germany (Ermisch 1956), Greece (Ermisch 1969), Hungary (Ermisch 1956), Italy (Ermisch 1956; Ruzzier 2013), Montenegro (**new country record**), Poland (Borowiec 1996), Russia (Ermisch 1956), Slovakia (Horák 2008), Spain (Batten 1976b), Switzerland (Méquignon 1946), Ukraine (Odnosum 2010).

#### Remarks.

*Variimorda mendax* inhabits various grassland and ruderal habitats. Adults are usually found feeding on the flowers of *Daucus carota*. Larvae and host plants remain unknown.

### Dellamora palposa

Taxon classificationAnimaliaColeopteraMordellidae

Normand, 1916

B012059D-938E-5078-BA0A-8A9C08C714CE

Dellamora palposa Normand, 1916: 285–286 [type locality: Téboursouk, Tunisia].

#### New records.

ITALY • 1 ♂; S. Angelo Muxaro, Sicilia; 23 Mar. 2017; L. Colacurcio leg.; E. Ruzzier det.; ERPC.

#### Distribution.

Cyprus (Horák 2008), Greece (Ermisch 1963c), Iran (Samin et al. 2016), Italy (**new country record**), Lebanon (Ermisch 1941), Mongolia (Samin et al. 2016), Morocco (Horák 2008), Portugal (Ermisch 1963c), Spain (Ermisch 1941), Tunisia (Normand 1916) Turkey (Horák 2008), Turkmenistan (Odnosum 1984).

#### Remarks.

Adults of *D. palposa* were reported to be found on the flowers of lotus (*Nelumbo*) (Normand 1916) and *Euphorbia* (Odnosum 1984). Larval stages and their bionomy remain unknown.

### Mordellistena (s. str.) dives

Taxon classificationAnimaliaColeopteraMordellidae

Emery, 1876

DC8EDA48-8BB6-59CB-B379-CFCA3EFE2744

Fig. 1B

Mordellistena (s. str.) dives Emery, 1876: 95 [type locality: Sarepta, Russia].

#### New records.

KAZAKHSTAN • 2 ♂♂; Aktjubinsk reg., Temir riv.; 27 May 1999; D. Selnekovič det.; DSPC.

#### Distribution.

Armenia (Horák 2008), Georgia (Horák 2008), Hungary (Kaszab 1979), Kazakhstan (**new country record**), Romania (Schilsky 1895), Russia (Emery 1876). Schilsky (1895) reported *M. dives* from “Süd-Ungarn: Mehadia” which is situated in present-day Romania.

#### Remarks.

Information about bionomy and host plants remain unknown.

### Mordellistena (s. str.) falsoparvula

Taxon classificationAnimaliaColeopteraMordellidae

Ermisch, 1956

A08D9E22-A135-5A0B-A9A9-EC639E1579AE

Mordellistena (s. str.) falsoparvula Ermisch, 1956: 281 [type locality: Mecklenburg, Germany].

#### New records.

BOSNIA AND HERZEGOVINA • 1 ♀; Bosnia; E. Bokor leg.; D. Selnekovič det.; HNHM • 2 ♀♀; Sarajevo; Apfelbeck leg.; D. Selnekovič det.; HNHM. MONTENEGRO • 1 ♂; Durdevica Tara Bridge env.; 43°08'49.4"N, 19°17'52.1"E, 3. June 2010; D. Selnekovič leg.; meadow; D. Selnekovič det.; DSPC.

#### Distribution.

Austria (Ermisch 1956), Belarus (Zemoglyadchuk 2007), Bosnia and Herzegovina (**new country record**), Bulgaria (Ermisch 1969a), Czech Republic (Ermisch 1956), Deutschland (Ermisch 1956), Estonia (Silfverberg 2004), France (Ermisch 1977), Georgia (Horák 2008), Hungary (Ermisch 1969a; Kaszab 1979), Italy (Ermisch 1969a; Ruzzier 2013), Kazakhstan (Odnosum 2010), Montenegro (**new country record**), Netherlands (Batten 1976a), Poland (Ermisch 1956; Borowiec 1996), Portugal (Horák 2008), Slovakia (Majzlan and Vidlička 2016), Switzerland (Borowiec 1996), Ukraine (Odnosum 2006), former Yugoslavia (Serbia and Montenegro) (Horák 2008).

#### Remarks.

This species inhabits xerothermophilous and mesophilous grasslands and ruderal vegetation where the adults feed on flowers of herbaceous plants, e.g., *Chrysanthemum*, *Cirsium*, *Achillea* (Borowiec 1996). The larva was described by Odnosum (1983, 2010) and reported to develop in stems of *Artemisia absinthium* and *A. vulgaris*. The first author reared adults from stem of Apiaceae plant infested by larvae in southern Slovakia.

### Mordellistena (s. str.) koelleri

Taxon classificationAnimaliaColeopteraMordellidae

Ermisch, 1956

CD53200F-3B0C-513E-8906-D4A3C3716B62

Mordellistena (s. str.) koelleri Ermisch, 1956: 289 [type locality: Hale, Lettiner Höhen, Germany].

#### New records.

ITALY • 1 ♂; Magredi di Cordenons, Pordenone, Friuli-Venezia Giulia; 46°01'50"N, 12°43'33"E; 02 June 2018; E. Ruzzier leg.; E. Ruzzier det.; ERPC • 1 ♂; Treviso, Veneto; 45°50'04"N, 11°44'47"E; 17 June 2018; E. Ruzzier leg.; E. Ruzzier det.; ERPC. MONTENEGRO • 2 ♂♂; Prokletije Mountains, Grebaje valley; 42°31'53"N, 19°47'36"E; 2 Aug. 2016; D. Selnekovič leg.; D. Selnekovič det.; DSPC.

#### Distribution.

Austria (Ermisch 1963b), Bosnia and Herzegovina (Ermisch 1963b), Bulgaria (Ermisch 1969b), Czech Republic (Ermisch 1963b), Denmark (Ermisch 1969a), France (Köstlin and Vogt 1971), Germany (Ermisch 1956), Hungary (Ermisch 1963b), Italy (**new country record**), North Macedonia (Ermisch 1969b), Poland (Borowiec 1996), Montenegro (**new country record**), Slovakia (Horák 1979), Spain (Horák 2008), Sweden (Kangas and Rutanen 1984), Switzerland (Köstlin and Vogt 1971), Ukraine (Odnosum 1993, 2010), former Yugoslavia (Horák 2008; without further information).

#### Remarks.

*Mordellistena koelleri* inhabits various grassland habitats from lowlands to highlands reaching up to 1700 m a.s.l. (Köstlin and Vogt 1971) The adults feed on flowers of herbaceous plants. It was observed by the authors on the subpannonian steppes and calcareous grasslands in Slovakia on flowers of *Tithymalus*, on a pastured montane meadow in Montenegro on *Daucus carota*, and a sandy steppe in Italy on flowers of *Daucus carota*. The larvae and host plants remain unknown. Specimens examined for the present study were compared with the type series deposited in SNSD.

### Mordellistena (s. str.) kraatzi kraatzi

Taxon classificationAnimaliaColeopteraMordellidae

Emery, 1876

F1B05CF4-B98E-50D3-B534-5761D9EC50EF

Mordellistena (s. str.) kraatzi kraatzi Emery, 1876: 91–92 [type locality: Sarepta, Russia].

#### New records.

MOROCCO • 2 ex.; 30 km NE Fez, Tissa env., Qued Leben riv.; 34°15'02"N, 4°45'54"W; 9 May 2015; E. Ezer leg.; D. Selnekovič det.; EEPC

#### Distribution.

Albania (Ermisch 1956), Azerbaijan (Horák 2008), Armenia (Horák 2008), Belarus (Zemoglyadchuk 2007), Bosnia and Herzegovina (Ermisch 1956), Bulgaria (Ermisch 1969b), Croatia (Ermisch 1956), Cyprus (Baudi di Selve 1877; Ermisch 1956), Czech Republic (Horák 1989), Georgia (Horák 2008), Germany (Ermisch 1956), Greece (Ermisch 1969b), Hungary (Schilsky 1895), Iran (Horák 2008), Iraq (Abdul-Rassoul 2010), Italy (Baudi di Selve 1877; Ruzzier 2013), Kazakhstan (Odnosum 2003), Kyrgyzstan (Horák 2008), North Macedonia (Ermisch 1969b), Morocco (**new country record**), Poland (Borowiec 1996), Portugal (Ermisch 1963c), Romania (Roubal 1936; Ermisch 1956), Russia (Emery 1876), Slovakia (Roubal 1936), Spain (Ermisch 1956), Switzerland (Baudi di Selve 1877), Syria (Ruzzier et al. 2017), Tajikistan (Odnosum 2003), Turkey (Ermisch 1956), Turkmenistan (Odnosum 2003), Ukraine (Odnosum 1993).

#### Remarks.

This species has a wide distributional range in Europe, North Africa and the western parts of Asia. It inhabits xerothermophilous grassland habitats where adults feed on flowers of various herbaceous plants (e.g., *Daucus carota*, *Tithymalus*). The larvae described by Odnosum (2010) feed on the stems of Asteraceae plants (e.g., *Arctium*, *Centaurea salonitana*).

### Mordellistena (s. str.) krujanensis

Taxon classificationAnimaliaColeopteraMordellidae

Ermisch, 1963

648941A8-5473-58D0-9739-10C0FB0023EA

Mordellistena (s. str.) krujanensis Ermisch, 1963b: 14, 17 [type locality: Kruja, Albania].

#### New records.

MONTENEGRO • 1 ♂, 1 ♀; Bar city; 42°06'N, 19°06'E; 19 June 2011; D. Selnekovič leg.; on flowers of *Daucus carota* in ruderal vegetation; D. Selnekovič det.; DSPC.

#### Distribution.

Ermisch (Ermisch 1963b) in the original description reported *M. krujanensis* from Albania and Bosnia and Herzegovina. *M. krujanensis* is recorded here for the first time from Montenegro.

#### Remarks.

The first author observed the adults on flowers of *Daucus carota* in ruderal vegetation along a roadside in Montenegro. The record represents a first report after the original description. The immature stages and host plants remain unknown.

### Mordellistena (s. str.) longicornis

Taxon classificationAnimaliaColeopteraMordellidae

Mulsant, 1856

04885546-E4F4-50FA-BCE7-51A174D1BF8E

Mordellistena (s. str.) longicornis Mulsant, 1856: 373–374 [type locality: France].

#### New record.

MOROCCO • 1 ♂; Col du Zad, Moyen Atlas Mts; 2100 m a.s.l.; 20 May 2015; E. Ruzzier det.; ERPC.

#### Distribution.

Armenia (Odnosum 2010), France (Mulsant 1856), Greece (Horák 2008), Morocco (**new country record**), Spain (Ermisch 1956; Plaza 1983), Ukraine (Odnosum 2005). Ermisch (1956) mentioned the occurrence of *M. longicornis* in North Africa without further information about the locality.

#### Remarks.

Adults have been reported feeding on flowers of *Thapsia villosa* in Spain (Plaza 1983). The immature stages are unknown.

### Mordellistena (s. str.) michalki

Taxon classificationAnimaliaColeopteraMordellidae

Ermisch, 1956

C7FD8233-B010-5E4B-BEC3-6E8BB53204C8

Mordellistena (s. str.) michalki Ermisch, 1956: 288 [type locality: Pernitz, Steirmark, Germany].

#### New records.

KYRGYZSTAN • 1 ♂; N Kirgizsky mountain range, Kara-Balta river; 1800 m a.s.l.; 28 June 1997; D. Selnekovič det.; DSPC.

#### Distribution.

Austria (Ermisch 1956), Bosnia and Herzegovina (Ermisch 1962), Croatia (Ermisch 1962), Czech Republic (Borowiec 1996), Germany (Ermisch 1962), Italy (Franciscolo 1995; Ruzzier 2013), Kazakhstan (Odnosum 1992), Kyrgyzstan (**new country record**), Russia (Odnosum 2010), Slovakia (Ermisch 1963b), Turkmenistan (Odnosum 2003).

#### Remarks.

This species inhabits steppes and xeric grasslands where adults feed on flowers of Apiaceae and Euphorbiaceae (e.g., *Seseli*, *Tithymalus*). The larvae and host plants remain unknown. Specimens examined for the present study were compared with the type series deposited in SNSD.

### Mordellistena (s. str.) olympica

Taxon classificationAnimaliaColeopteraMordellidae

Ermisch, 1965

FF4D22E4-845C-5A7D-AAB0-2AF7FF7C709F

Mordellistena (s. str.) olympica Ermisch, 1965: 265, 268–269 [type locality: Morea, Olymp env., Greece].

#### New records.

CYPRUS • 6 ♂♂, 2 ♀♀; Skoulli village env.; 34°58'17"N, 32°27'02"E; 24 Apr. 2018; D. Selnekovič leg.; in ruderal vegetation along the road; D. Selnekovič det.; DSPC • 2 ♂♂, 2 ♀♀; Skoulli village env.; 34°58'05"N, 32°26'46"E; 24 Apr. 2018; D. Selnekovič leg.; ruderal vegetation along the field margin, on the flowers of *Daucus carota*; D. Selnekovič det.; DSPC. MONTENEGRO • 2 ♂♂, 1 ♀; Bar city, Stari Bar; 42°05'31"N, 19°07'58"E, 19 June 2011; D. Selnekovič leg.; D. Selnekovič det.; DSPC • 3 ♂♂, 1 ♀; Bar city, Volujica hill; 42°04'16"N, 19°06'10"E; 20 June 2011; dry grasslands along the sea coast; D. Selnekovič det.; DSPC • 26 ♂♂, 17 ♀♀; Virpazar env.; 42°14'40"N, 19°05'36"E; 30 m a.s.l.; 21 June 2011; D. Selnekovič leg.; D. Selnekovič det.; DSPC.

#### Distribution.

Bulgaria (Ermisch 1965), Cyprus (**new country record**), Greece (Ermisch 1965), North Macedonia (Ermisch 1965), Montenegro (**new country record**), Turkey (Horák 2008) and former Yugoslavia (Horák 2008; without further information).

#### Remarks.

**Figure 2.**
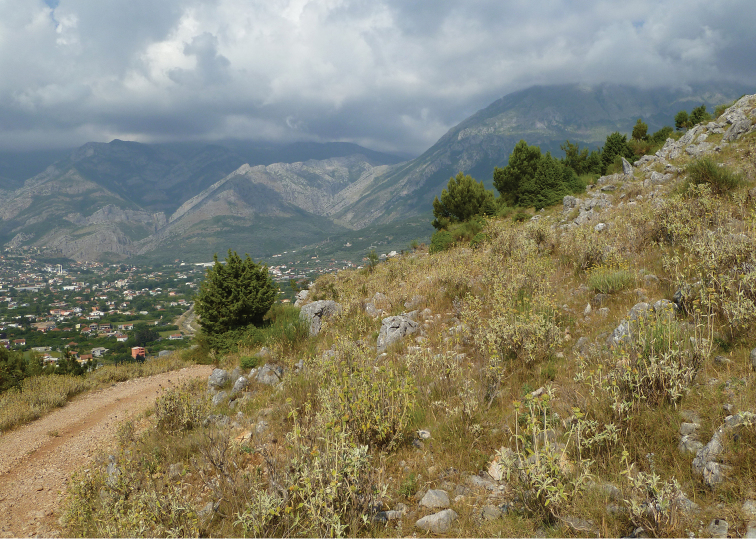
Mediterranean xeric grasslands with flowering *Helichrysum* on Volujica hill in Montenegro, 42°04'29.0"N, 19°06'11.8"E. *Variimorda caprai* (Franciscolo, 1951), *Mordellistena olympica* Ermisch, 1965 and *Mordellistenula longipalpis* Ermisch, 1965 recorded from this locality are new to Montenegro.

**Figure 3.**
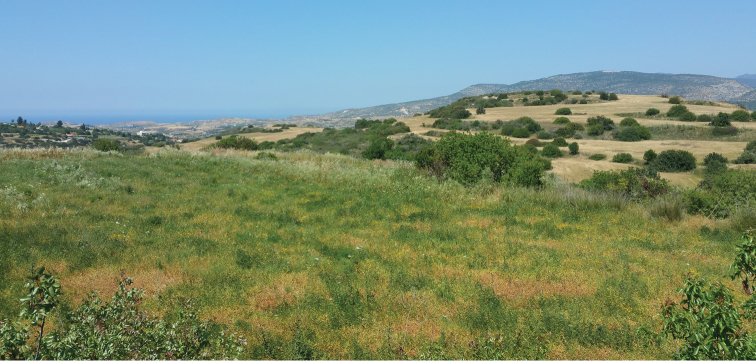
Ruderal vegetation near Skoulli village in Cyprus, 34°58'05"N, 32°26'46"E. *Mordellistena olympica* Ermisch, 1965 and *M. tarsata* Mulsant, 1856 recorded from this locality are new to Cyprus.

The first author collected adults in Mediterranean xeric grasslands and ruderal vegetation along roadsides and field margins in Bulgaria, Montenegro (Fig. 2) and Cyprus (Fig. 3). The specimens were feeding on flowers of *Daucus carota*. The immature stages and host plants remain unknown. Specimens examined for the present study were compared with the type specimens deposited in SNSD.

### Mordellistena (s. str.) thuringiaca

Taxon classificationAnimaliaColeopteraMordellidae

Ermisch, 1963

C85E9C0A-F752-50FF-AC46-50FCF78E390C

Mordellistena (s. str.) thuringiaca Ermisch, 1963b: 23–24 [type locality: Kyffhäusers bei Frankenhausen, Germany].

#### New records.

BULGARIA • 1 ♂; Kresna, Struma banka; 24 May–4 June 1976; K. Majer leg.; D. Selnekovič det.; SNMB • 1 ♂; Lilyanovo village env.; 41°37'23"N, 23°19'41"E; 26 June 2015; D. Selnekovič leg.; D. Selnekovič det.; DSPC. MONTENEGRO • 1 ♂; Virpazar env.; 42°14'40"N, 19°05'36"E; 30 m a.s.l.; 21 June 2011; D. Selnekovič leg.; D. Selnekovič det.; DSPC. SLOVAKIA • 2 ♂♂; Chľaba village env.; 47°49'52.6"N, 18°49'55.3"E; 8 June 2011; D. Selnekovič leg.; meadow, on flowers of *Tithymalus*; D. Selnekovič det.; DSPC • 3 ♂♂; Kamenín village, Kamenínske slanisko; 47°52'43.2"N, 18°38'46.5"E; 10 June 2011; D. Selnekovič leg.; halophile grassland, on flowers of *Galium vernum*; D. Selnekovič det.; DSPC • 1 ♂; Podhoroď village, Papratný vrch; 48°49'07"N, 22°18'24"E; 15 July 2011; D. Selnekovič leg.; meadow, on flowers of *Daucus carota*; D. Selnekovič det.; DSPC • 1 ♂; Silická Brezová village env.; 48°31'30"N, 20°29'07"E; 3 July 2014; D. Selnekovič leg.; dry grassland; D. Selnekovič det.; DSPC • 1 ♂; Tvrdošovce village env.; 48°06'01"N, 18°01'59"E; 26 July 2016; D. Selnekovič leg.; halophile grassland; on the flowers of *Daucus carota*; D. Selnekovič det.; DSPC • 1 ♂; Banský Studenec village env.; 48°26'14"N, 18°59'38"E; 25 June 2017; D. Selnekovič leg.; meadow; D. Selnekovič det.; DSPC. SPAIN • 3 ♂♂ [specimens identified by Ermisch as an undescribed species *M. balearica*]; Son Españolet; 26 May–9 June 1958; R. López leg.; D. Selnekovič det.; SNSD.

#### Distribution.

Austria (Köstlin and Vogt 1971), Belarus (Zemoglyadchuk 2007), Bulgaria (**new country record**), France (Ermisch 1963b), Germany (Ermisch 1963b), Hungary (Ermisch 1963b), Italy (Ruzzier 2013), Kazakhstan (Odnosum 1992a), Montenegro (**new country record**), Poland (Borowiec 1996), Russia (Odnosum 1992b), Slovakia (**new country record**), Spain (**new country record**), Switzerland (Köstlin and Vogt 1971), Turkmenistan (Odnosum 2003), Ukraine (Odnosum 2010). Records from the Russian Far East (Odnosum 1992b) need to be revised.

#### Remarks.

**Figure 4.**
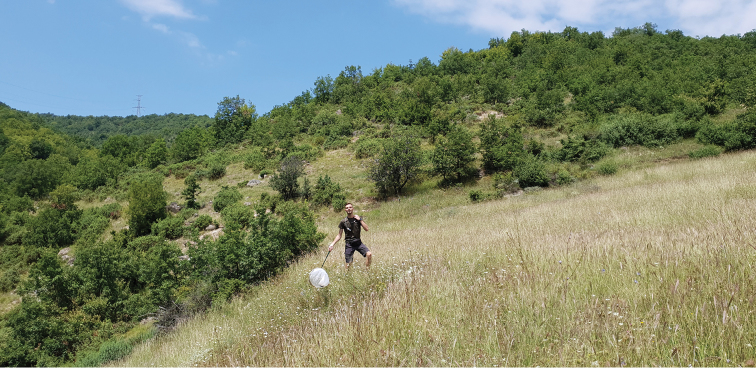
Xeric grassland near Lilyanovo village in Pirin Mountains in Bulgaria, 41°37'23"N, 23°19'41"E. *Mordellistena thuringiaca* Ermisch, 1963 recorded from this locality is new to Bulgaria.

It is an infrequently found species inhabiting various grassland habitats. The first author observed adults on xerothermophilous (Fig. 4) and mesophilous grasslands on flowers of herbaceous plants (e.g., *Daucus carota*, *Tithymalus*, *Galium*). The immature stages and host plants remain unknown. Specimens examined for the present study were compared with the type series deposited in SNSD.

### Mordellistena (s. str.) tarsata

Taxon classificationAnimaliaColeopteraMordellidae

Mulsant, 1856

C88A177E-59D5-59F8-BB17-0341DBEB1483

Mordellistena (s. str.) tarsata Mulsant, 1856: 381 [type locality: Lyon env., France].

#### New records.

CYPRUS • 2 ♂♂; Skoulli village env.; 34°58'17"N, 32°27'02"E; 24 Apr. 2018; D. Selnekovič leg.; in ruderal vegetation along the road; D. Selnekovič det.; DSPC. NORTH MACEDONIA • 1 ♀; Nichpur village env.; 41°43'15"N, 20°40'06"E; 930 m a.s.l.; 19 Aug. 2018; D. Selnekovič leg.; river valley; on the flowers of *Daucus carota*; D. Selnekovič det.; DSPC.

#### Distribution.

Albania (Ermisch 1956), Algeria (Csiki 1915), Austria (Ermisch 1956), Bosnia and Herzegovina (Ermisch 1956), Bulgaria (Ermisch 1956), Croatia (Schilsky 1895; Ermisch 1956), Cyprus (**new country record**), Czech Republic (Ermisch 1956), France (Mulsant 1856), Georgia (Ermisch 1956), Germany (Csiki 1915; Ermisch 1956), Greece (Ermisch 1969b), Hungary (Ermisch 1956) Italy (Emery 1876; Baudi di Selve 1878; Ruzzier 2013), Kazakhstan (Odnosum 1992a), Kyrgyzstan (Odnosum 2003), North Macedonia (**new country record**), Mongolia (Odnosum 1992b), Romania (Ermisch 1956; as Hungary: Mehadia), Russia (Odnosum 1992b), Slovakia (Horák 1989), Spain (Ermisch 1956; Plaza 1983), Switzerland (Ermisch 1963b), Turkey (Ermisch 1956), Turkmenistan (Odnosum 2003), Ukraine (Odnosum 1993, 2010), former Yugoslavia (Serbia and Montenegro; Horák 2008; without further information).

#### Remarks.

*Mordellistena tarsata* has a wide distributional range across the Palearctic realm. It inhabits dry grasslands and ruderal vegetation (Fig. 3) where adults feed on flowers of various herbaceous plants, e.g., *Daucus carota*, *Rubus fruticosus*, *Achillea millefolium*, *Filipendula ulmaria* (Ermisch 1963b), *Heracleum spondylium* (Ermisch 1963b), *Thapsia villosa* (Plaza 1983), *Ruta montana* (Plaza 1983). Although *M. tarsata* is widely distributed, it is not a common species, and usually, only a few individuals are found in a particular locality. The immature stages and their host plants remain unknown.

### Mordellistenochroa fallaciosa

Taxon classificationAnimaliaColeopteraMordellidae

(Ermisch, 1969)

66260768-B0F4-5C42-93A3-BB51AD400F39

Mordellistena (s. str.) fallaciosa Ermisch, 1969c: 110–111 [type locality: “Grado bei Triest”, Italy].
Mordellistenochroa fallaciosa : Horák (1990: 141).

#### New records.

ITALY • 1 ♂; Cesenatico (dry canal), Forlì-Cesena Emilia-Romagna; 9 June 2012; L. Colacurcio leg., E. Ruzzier det.; ERPC.

#### Distribution.

Hungary (Merkl and Németh 2008), Italy (Ermisch 1969c), Switzerland (Horák 2008).

#### Remarks.

Up to present, *M. fallaciosa* is known only from several localities in Italy, Hungary and Switzerland (Ermisch 1969c; Merkl and Németh 2008; Horák 2008). Although this species was originally described from Italy by Ermisch (1969c), we provide here another record of this rare species, increasing the information about its distribution in the country. The immature stages and their host plants remain unknown.

### Mordellistenula longipalpis

Taxon classificationAnimaliaColeopteraMordellidae

Ermisch, 1965

EABACA9C-E6C1-575F-B9DE-332DA4B78C34

Fig. 5A

Mordellistenula longipalpis Ermisch, 1965: 256–259 [type locality: Belgrader wald, Turkey].

#### New records.

MONTENEGRO • 8 ♂♂, 5 ♀♀; Bar city env., Volujica hill; 42°04'16"N, 19°6'10"E; 20 June 2011; D. Selnekovič leg.; dry grassland along the seashore, on the flowers of *Helichrysum*; D. Selnekovič det.; DSPC.

#### Distribution.

Armenia (Horák 2008), Azerbaijan (Horák 2008), Bulgaria (Ermisch 1969b), Greece (Ermisch 1969b), Kazakhstan (Odnosum 2003), North Macedonia (Horák 2008), Montenegro (**new country record**), Turkey (Ermisch 1965), Ukraine (Odnosum 2010), former Yugoslavia (Horák 2008, without specification).

#### Remarks.

This species is known from the Balkans and western Asia. It inhabits dry grasslands where adults feed on flowers of herbaceous plants. The first author observed adults on Mediterranean xeric grassland along the seashore on flowers of *Helichrysum* in Montenegro (Fig. 2), and in ruderal vegetation along roadsides on flowers of *Daucus carota* in Bulgaria. The immature stages and their host plants remain unknown.

**Figure 5.**
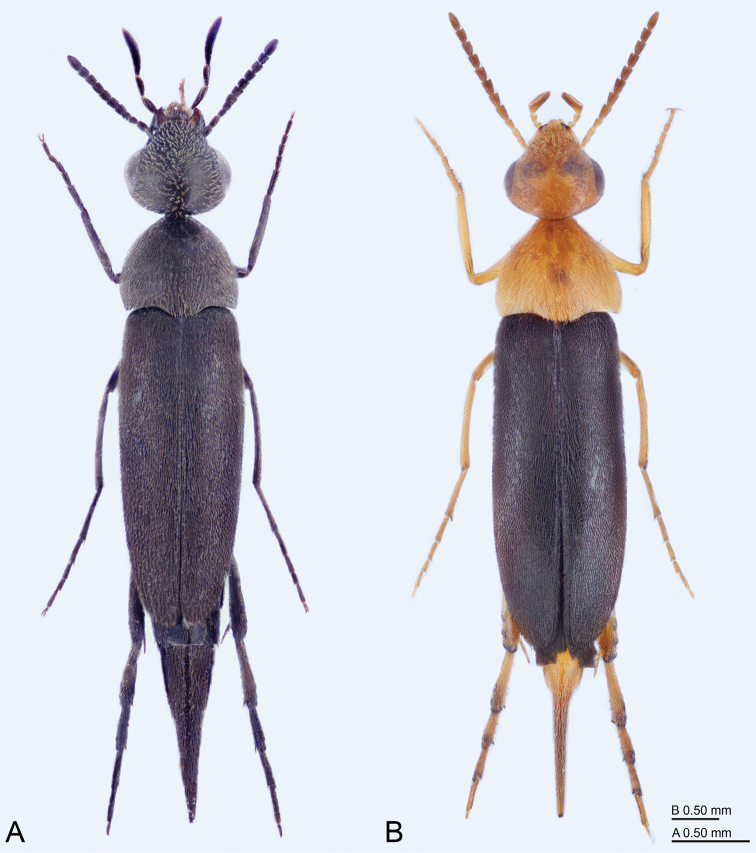
**A***Mordellistenula longipalpis* Ermisch, 1965, male **B***Mordellochroa milleri* (Emery, 1876), male.

### Mordellochroa milleri

Taxon classificationAnimaliaColeopteraMordellidae

(Emery, 1876)

E8D70F7D-5054-547F-838B-D7DB86450FC2

Fig. 5B

Mordellistena (Mordellochroa) milleri Emery, 1876: 80, 83 [type locality: Mehadia, Romania].
Mordellistena (Tolida) milleri : Reitter (1911: 376).
Tolida milleri : Ermisch (1941: 717).
Mordellochroa milleri : Ermisch (1950: 78–79).

#### New records.

ITALY • 1 ♀; Lago I Piani, Rincine, Londa, Firenze, Toscana; 43°52'55"N, 11°35'47"E; C. Massarone leg.; E. Ruzzier det.; ERPC • 1 ♀; Emilia-Romagna, Castel D’Aiano, Spe Mountain, Bologna; 5 July 2014; L. Colacurcio leg.; E. Ruzzier det.; ERPC • 1 ♀; Emilia-Romagna, M. S. Pietro – S. Martino, Bologna; 25–29 May 2016; L. Colacurcio leg.; E. Ruzzier det.; ERPC • 2 ♀♀; Emilia-Romagna, M. S. Pietro – S. Martino, Bologna; 1–10 July 2016; L. Colacurcio leg.; E. Ruzzier det.; ERPC • 1 ♀; Emilia-Romagna, M. S. Pietro – S. Martino, Bologna; 11–20 July 2016; L. Colacurcio leg.; E. Ruzzier det.; ERPC • 1 ♀; Emilia-Romagna, M. S. Pietro – S. Martino, Bologna; 18 May 2017; L. Colacurcio leg.; E. Ruzzier det.; ERPC.

#### Distribution.

Austria (Reitter 1911), Azerbaijan (Odnosum 1996), Czech Republic (Horák 2008), France (Sainte-Claire Deville 1936), Hungary (Horák 2008), Italy (**new country record**), Poland (Kubisz 2000), Romania (Emery 1876), Slovakia (Roubal 1936), Slovenia (Horák 2008), Spain (Viñolas et al. 2009), Switzerland (Sanchez et al. 2015), Ukraine (Reitter 1911; Odnosum 1996).

#### Remarks.

*Mordellochroa milleri* usually occurs in beech, oak or floodplain forests, and on its margins, but it was also reported from coniferous forest with spruces and pines in Bialowieza National Park, Poland (Kubisz 2000). Adults were found feeding on flowers of Apiaceae (e.g., *Libanotis montana*) and Brassicaceae (*Cardaria draba*) plants. Larvae feed in dead wood.

## Discussion

The Palearctic fauna of Mordellidae consists of approximately 700 described species (Horák 2008; Odnosum 2009; Takakuwa 2010; Horák et al. 2012; Ruzzier and Kovalev 2016; Tsuru 2017; Selnekovič and Kodada 2019). The most recent comprehensive catalogue of Palearctic Mordellidae was provided by Horák (2008). Since then several new country-level records were published in the catalogues focused on smaller geographical areas (Abdul-Rassoul 2010; Serrahima 2011; Horák et al. 2012; Ruzzier 2013; Ruzzier et al. 2017) or as single-species records (Horák and Háva 2008; Ferenca and Tamutis 2009; Odnosum 2009; Viñolas et al. 2009; Diéguez Fernández 2010; Sanchez et al. 2015; Viñolas et al. 2016; Selnekovič and Kodada 2019). Although the distribution of Mordellidae species has been studied mostly in the western Palearctic, the recent fieldwork combined with efforts to re-examine the material deposited in major European collections have revealed 22 new country-level records for 16 species. Such results suggest that our knowledge of the distribution of Mordellidae species can be still considered poor and that continuous sampling effort will lead to further new and interesting records.

Furthermore, huge gaps exist in our knowledge of the bionomy of these beetles. Information about habitat preferences and host plants is scarce. Most of the larval descriptions and host plant records were provided by V. K. Odnosum (e.g., Odnosum 1983, 1985, 2010; Odnosum and Litvin 2009). Despite his and other authors’ great effort, the number of species for which larval stages are described is relatively low. The general lack of information makes it extremely difficult to identify the larvae based solely on their morphology. The use of DNA barcoding should simplify the species identifications in the future and reveal new information about host-plant relationships and habitat preferences.

Each published record represents a small step to better understanding the distribution and bionomy of mordellid beetles, and their role in the ecosystems. Such information is becoming still more critical, especially in the current age of global environmental changes and loss of natural habitats.

## Supplementary Material

XML Treatment for Variimorda (Galeimorda) caprai

XML Treatment for Variimorda (s. str.) mendax

XML Treatment for Dellamora palposa

XML Treatment for Mordellistena (s. str.) dives

XML Treatment for Mordellistena (s. str.) falsoparvula

XML Treatment for Mordellistena (s. str.) koelleri

XML Treatment for Mordellistena (s. str.) kraatzi kraatzi

XML Treatment for Mordellistena (s. str.) krujanensis

XML Treatment for Mordellistena (s. str.) longicornis

XML Treatment for Mordellistena (s. str.) michalki

XML Treatment for Mordellistena (s. str.) olympica

XML Treatment for Mordellistena (s. str.) thuringiaca

XML Treatment for Mordellistena (s. str.) tarsata

XML Treatment for Mordellistenochroa fallaciosa

XML Treatment for Mordellistenula longipalpis

XML Treatment for Mordellochroa milleri

## References

[B1] Abdul-Rassoul MS (2010) Tumbling flower beetles (Coleoptera, Mordellidae) of Iraq. Bulletin of the Iraq Natural History Museum 11(2): 1–5.

[B2] Batten R (1976a) De Nederlandse soorten van de keverfamilie Mordellidae. Zoölogische Bijdragen 19: 3–37.

[B3] Batten R (1976b) Mordellidae (Coleoptera) from the South of France and the Pyrenees. Entomologische Berichten 36: 164–171.

[B4] Baudi di Selve F (1878) Coleotteri eteromeri esistenti nelle collezioni del R. Museo zoologico di Torino ed in altre italiane. Eteromeri delle famiglie susseguenti a quella dei tenebrioniti nei limiti della fauna europaea e circummediterranea. Atti della Reale Accademia delle Scienze di Torino 13: 765–866, 1027–1183.

[B5] Borowiec L (1996) Mordellidae, Miastkowate (Insecta: Coleoptera). Fauna Polski, Vol. 18. Muzeum i Instytut Zoologii, Warszawa, 190 pp.

[B6] Csiki E (1915) Pars 63: Mordellidae. In: Junk W Schenkling S (Eds) Coleopterorum Catalogus.W. Junk, Berlin, 1–51.

[B7] Diéguez Fernández JM (2010) Primera cita de *Curtimorda maculosa* (Neazen 1794) para la Península Ibérica (Coleoptera: Mordellidae). Arquivos Entomolóxicos 4: 15–16.

[B8] Emery MC (1876) Essai monographique sur les Mordellides de l’Europe et des contrées limitrophes. L’Abeille: Journal d’Entomologie 14: 1–128.

[B9] Ermisch K (1941) Tribus Mordellistenini (Col. Mordell.). Mitteilungen der Münchener Entomologischen Gesellschaft 31: 710–726.

[B10] Ermisch K (1956) Mordellidae. In: Horion A (Ed.) Faunistik der mitteleuropäischen Käfer.Band 5: Heteromera. Entomologische Arbeiten aus dem Museum G. Frey Tutzing bei München, München, 269–328.

[B11] Ermisch K (1962) 17. *Mordellistena breddini* n. sp. aus dem Siebengebirge und der Eifel. Decheniana 10: 183–186.

[B12] Ermisch K (1963a) Die Mordelliden der Insel Cypern (Coleoptera, Heteromera, Mordellidae). Notulae Entomologicae 43: 49–67.

[B13] Ermisch K (1963b) Neue Mordelliden (Heteromera, Mordellidae) aus Deutschland und Nachträge zur Faunistik der Mitteleuropaischen Mordelliden. Entomologische Blätter 59: 1–36.

[B14] Ermisch K (1963c) Beitrag zur Mordelliden-Fauna Portugals (Coleopt. Heteromera, Mordellidae). Notulae Entomologicae 42: 14–21.

[B15] Ermisch K (1965) Neue Mordelliden von der Balkanhalbinsel (Coleoptera, Mordellidae). Reichenbachia 5(30): 251–272.

[B16] Ermisch K (1966) Neue westpaläarktische *Mordellistena*-Arten (Coleoptera-Heteromera-Mordellidae). Entomologische Blätter 62(1): 30–39.

[B17] Ermisch K (1969a) 79. Familie: Mordellidae. In: Freude H Harde KW Lohse GA (Eds) Die Käfer Mitteleuropas.Band 8, Teredilia, Heteromera, Lamellicornia. Goecke & Evers, Krefeld; G. Fischer, Jena, Stuttgart, 160–196.

[B18] Ermisch K (1969b) Ergebnisse der Albanien-Expedition 1961 des Deutschen Entomologischen Institutes, 76. Beitrag, Coleoptera: Mordellidae. Beiträge zur Entomologie 19(7/8): 845–859.

[B19] Ermisch K (1969c) Neue Mordelliden aus Europa, Nordafrika und dem Nahen Osten. Entomologische Blätter 65(2): 104–115.

[B20] Ermisch K (1977) Die *Mordellistena*-Arten Ungarns und benachbarter Gebiete sowie Beschreibung einer neuen *Hoshihananomia*-Art aus Siebenbürgen. Folia Entomologica Hungarica (New Series) 30: 151–177.

[B21] Ferenca R Tamutis V (2009) Data on seventeen beetle (Coleoptera) species new for Lithuanian fauna. New and Rare for Lithuania Insect Species 21: 32–39.

[B22] Franciscolo ME (1951) Una nuova *Mordella* dell’Albania. Bollettino della Società Entomologica Italiana 81: 7–9.

[B23] Franciscolo ME (1995) Famm. Mordellidae. In: Angelini F, Audisio P, Bologna MA, de Biase A, Franciscolo ME, Nardi G, Ratti E, Zampetti MF, ColeopteraPolyphaga XII (Heteromera escl. Lagriidae, Alleculidae, Tenebrionidae). In: Minelli A Ruffo S La Posta A (Eds) Checklist delle specie della fauna italiana, fasc.58. Ed. Calderini, Bologna, 11–13.

[B24] Heyden L Reitter E Weise J (1906) Mordellidae. In: Catalogus coleopterorum Europae, Caucasi et Armeniae Rossicae. Editio secunda. Friedländer & Sohn, Berlin, Paskau, Caen, 453–458.

[B25] Hill GF (1922) A new species of *Mordellistena* (Coleoptera, fam. Mordellidae) parasitic on termites. Proceedings of the Linnean Society of New South Wales 47: 346–347.

[B26] Horák J (1979) Faunistic records from Czechoslovakia. Mordellidae. Acta Entomologica Bohemoslovaca 66: 346.

[B27] Horák J (1985) Ergebnisse der tschechoslowakisch-iranischen entomologischen Expeditionen nach Iran 1970, 1973 und 1977, Coleoptera: Mordellidae 1 (Stenaliini, Mordellini). Entomologische Abhandlungen 49(1): 1–25.

[B28] Horák J (1989) Faunistic records from Czechoslovakia. Coleoptera, Mordellidae. Acta Entomologica Bohemoslovaca 86: 477–478.

[B29] Horák J (1990) Typenrevision einiger wenig bekanntner Arten aus der Gattung *Mordellistena* Costa (Insecta, Coleoptera: Mordellidae). Entomologische Abhandlungen 53(9): 125–142.

[B30] Horák J (2008) Family Mordellidae Latreille, 1802. In: Löbl I Smetana A (Eds) Catalogue of Palaearctic Coleoptera.Vol. 5. Tenebrionoidea. Apollo Books, Stenstrup, 87–105.

[B31] Horák J Farkač J Nakládal O (2012) Mordellidae (Coleoptera) from Socotra Island. Acta Entomologica Musei Nationalis Pragae 52 (Suppl. 2): 253–268.

[B32] Horák J Háva J (2008) Faunistic records from the Czech Republic – 259. Klapalekiana 44: 236.

[B33] Kangas E Rutanen I (1984) Four *Mordellistena* species (Coleopetra: Mordellidae) new to Sweden. Entomologisk Tidskrift 105: 99–101.

[B34] Kaszab Z (1979) Felemás lábfejízes bogarak II. – Heteromera II. In: Magyarország állatvilága (Fauna Hungariae), IX, 2. Akadémiai Kiadó, Budapest, 105 pp.

[B35] Köstlin R Vogt H (1971) Beitrag zur Mordellidenfauna. Mitteilungen des Entomologischen Vereins Stuttgart 6: 35–74.

[B36] Kubisz D (2000) *Mordellochroa milleri* Emery (Mordellidae), *Anaspis bohemica* Schilsky (Scraptiidae) and *Corticeus bicoloroides* (Roubal) (Tenebrionidae) – Tenebrionidea (Coleoptera) new to the Polish fauna. Wiadomości Entomologiczne 19(1): 9–14.

[B37] Lawrence JF Ślipiński A (2010) Mordellidae Latreille, 1802. In: Leschen RAB Beutel RG Lawrence JF (Eds) Handbook of Zoology, Coleoptera, Beetles, Morphology and Systematics (Elateroidea, Bostrichiformia, Cucujiformia partim), Volume 2.Walter de Gruyter, Berlin-New York, 533–537. 10.1515/9783110911213.533

[B38] Majzlan O Vidlička L (2016) Specific diversity of beetles (Coleoptera) near cowsheds. Entomofauna Carpathica 28(1): 1–13.

[B39] Méquignon A (1946) Contribution à l’étude des mordellides paléarctiques. Revue Française d’Entomologie 13: 52–76.

[B40] Merkl O Németh T (2008) Notes on and further new species of the beetles in the Hungarian fauna (Coleoptera). Folia Entomologica Hungarica 69: 165–172.

[B41] Mulsant E (1856) Histoire des coléoptères de France. Barbipalpes, longipèdes, latipennes. Annales de la Société Linnéenne de Lyon (New Series) 3: 193–544.

[B42] Normand H (1916) Nouveaux coléoptères de la faune tunisienne. Bulletin de la Société Entomologique de France 1916: 283–287.

[B43] Odnosum VK (1983) Morfologiya litschinki zhuka-gorbatki *Mordellistena falsoparvula*. Vestnik Zoologii 1983(5): 82–84.

[B44] Odnosum VK (1984) Novyj dlya fauny SSSR rod *Dellamora* Normand (Coleoptera, Mordellidae) iz Turkmenii. Vestnik Zoologii 1984: 22.

[B45] Odnosum VK (1985) K diagnostike litschinok Zhukov-gorbatok (Coleoptera, Mordellidae) fauny Ukrainy. Vestnik Zoologii 1985(2): 24–28.

[B46] Odnosum VK (1992a) Zhuki-gorbatki (Coleoptera, Mordellidae) fauny Kazakhstana. Vestnik Zoologii 1992(6): 32–39.

[B47] Odnosum VK (1992b) Sem. Mordellidae – gorbatki ili shiponoski. In: Ler PA (Ed.) Opredelitel nasekomych Dalnego Vostoka SSSR v shestykh tomakh.Tom III. Zhestokrylye, ili zhuki. Chast 2. Nauka, Saint Petersburg, 517–526.

[B48] Odnosum VK (1993) Subfamily Mordellinae mordellid beetles (Coleoptera, Mordellidae) of the Ukrainian fauna. Communication 2. Vestnik Zoologii 1993(6): 20–28.

[B49] Odnosum VK (1996) Mordellid-beetles of the genus *Mordellochroa* (Coleoptera, Mordellidae) of the East Palaearctics. Vestnik Zoologii 1996(6): 47–52.

[B50] Odnosum VK (2003) Mordellid Beetles (Coleoptera, Mordellidae) in the Fauna of Kazakhstan and Middle Asia. Communication 2. Vestnik Zoologii 37(4): 33–49.

[B51] Odnosum VK (2005) Tumbling flower beetles (Coleoptera: Mordellidae) of the central and eastern Europe fauna. Communication 2. Subfamily Mordellinae. Tribe Mordellistenini. The Kharkov Entomological Society Gazette 11(1–2): 93–112.

[B52] Odnosum VK (2006) Mordellid beetles of the *Mordellistena parvula* group (Coleoptera, Mordellidae) in the fauna of Ukraine. Vestnik Zoologii 40(4): 311–319.

[B53] Odnosum VK (2009) Review of Mordellid Beetles of the *Mordellistena confinis* group (Coleoptera, Mordellidae). Vestnik Zoologii 43(1): 3–14. 10.2478/v10058-009-0023-9

[B54] Odnosum VK (2010) Vypusk 9, Zhuki-Gorbatki (Coleoptera, Mordellidae). Fauna Ukrainy, tom 19, Zhestokrylye. Naukova Dumka, Kiev, 263 pp.

[B55] Odnosum VK Litvin O (2009) Description of *Mordellistena parvuliformis* larva (Coleoptera, Mordellidae). Vestnik Zoologii 43(6): 18–20. 10.2478/v10058-009-0023-9

[B56] Plaza E (1983) Mordellidae (Col.) de la provincial de Madrid. Actas del I Congreso Iberico de Entomologia 1983: 567–577.

[B57] Plaza E (1985) Las especies españolas de *Mordellistena* Costa del grupo *episternalis* (Col., Mordellidae). Eos: Revista Española de Entomología 61: 275–292.

[B58] Reitter E (1911) Fauna Germanica. Die Käfer des Deutschen Reiches. Nach der analytischen Methode bearbeitet. Band 3. K. G. Lutz, Stuttgart, 436 pp.

[B59] Roubal J (1936) Katalog Coleopter (brouků) Slovenska a Podkarpatské Rusi na základě bionomickém a zoogeografickém a spolu systematický doplněk Ganglbauerových “Die Käfer Mitteleuropas” a Reitterovy “Fauna germanica”. Vol. II. Slovanský ústav v nakladatelství Orbis, Bratislava, 434 pp.

[B60] Ruzzier E (2013) Taxonomic and faunistic notes on Italian Mordellidae (ColeopteraTenebrionoidea) with redescription of *Falsopseudotomoxia argyropleura* (Franciscolo, 1942) n. comb. Bollettino della Società Entomologica Italiana 145(3): 103–115. 10.4081/BollettinoSEI.2013.103.

[B61] Ruzzier E Kovalev AV (2016) First record of *Calycina* Blair, 1922 (Coleoptera, Mordellidae) in the Russian Far East with description of a new species. Zootaxa 4103(1): 075–078. 10.11646/zootaxa.4103.1.9 27394617

[B62] Ruzzier E Ghahari H Horák J (2017) A checklist of the Iranian Mordellidae (Coleoptera: Tenebrionidea). Zootaxa 4320(1): 146–158. 10.11646/zootaxa.4320.1.8

[B63] Sainte-Claire Deville J (1936) Catalogue raisonné des Coléoptères de France. (Complete et publie par Mequignon A.). L’Abeille: Journal d’Entomologie 36(2): 161–264.

[B64] Samin N Háva J Kubisz D (2016) A contribution to the knowledge of some families of Coleoptera (Insecta) from Iran. Arquivos Entomolóxicos 15: 29–38.

[B65] Sanchez A Chittaro Y Monnerat Ch. (2015) Coléoptères nouveaux ou redécouverts pour la Suisse ou l’une de ses régions biogéographiques. Entomo Helvetica 8: 119–132.

[B66] Schilsky J (1895) Die Käfer Europa’s. Nach der Natur Beschrieben von Dr. H. C. Küster und Dr. G. Kraatz Fortgesetzt von J. Schilsky, Vol 31. Bauer und Raspe (Emil Küster), Nürnberg 1–100. [viii + no. taxa]

[B67] Selnekovič D Kodada J (2019) Taxonomic revision of *Mordellistena hirtipes* species complex with new distribution records (Insecta, Coleoptera, Mordellidae). ZooKeys 854: 89–118. 10.3897/zookeys.854.32299 31231159 PMC6579795

[B68] Serrahima I (2011) Catálogo provisional de los Mordellidae (Coleoptera) de Cataluña (España). Boletín de la Sociedad Entomológica Aragonesa 48: 375–381.

[B69] Silfverberg H (2004) Enumeratio nova Coleopterorum Fennoscandiae, Daniae et Baltiae. Sahlbergia 9: 1–111.

[B70] Takakuwa M (2000) A taxonomic study of the mordellid subgenus Stenoglipa (Coleoptera, Mordellidae). Bulletin of the Kanagawa Prefectural Museum (Natural Science) 29: 53–105.

[B71] Takakuwa M (2010) Two unexpected new species of the genus *Variimorda* (Coleoptera, Mordellidae) from the Ogasawara Islands. Elytra, Tokyo 38(2): 193–200.

[B72] Tooker JF Hanks LM (2004) Trophic position of the endophytic beetle, *Mordellistena aethiops* Smith (Coleoptera: Mordellidae). Environmental Entomology 33(2): 291–296. 10.1603/0046-225X-33.2.291

[B73] Tsuru TK (2017) A new species of the genus *Mordellina* (Coleoptera, Mordellidae, Mordellistenini) from Okinawa Island, southwestern Japan. Elytra, New Series 7(2): 305–311.

[B74] Viñolas A Muñoz J Soler J (2009) Noves o interresants citacions de coleòpters per a Catalunya (Parc Natural del Montseny) i per a la peninsula Ibèrica (Coleoptera) (3a nota). Orsis 24: 159–167.

[B75] Viñolas A Muñoz-Batet J Soler J (2016) Noves o interessants localitzacions d’espècies de coleòpters per a la península Ibèrica i illes Canàries (Coleoptera). Butlletí de la Institució Catalana d’Història Natural 80: 101–112.

[B76] Zemoglyadchuk AV (2007) Species composition and biotopical distribution of the mordellid beetles (Coleoptera, Mordellidae) of the Belarus fauna. Bulletin of Moscow Society of Naturalists, Biological series 112(2): 14–17.

